# Differential Ophthalmological Profile in Patients with Coronary Artery Disease Coexisting with Type 2 Diabetes Mellitus: Elevated Tear Cytokine Concentrations

**DOI:** 10.3390/jcm13164906

**Published:** 2024-08-20

**Authors:** Rafael Jiménez-López, José Lorenzo Romero-Trevejo, Lourdes Fernández-Romero, Laura Martín-Chaves, Miguel Romero-Cuevas, Ana Isabel Molina-Ramos, María José Sánchez-Quintero, Mora Murri, Francesco Costa, Vicente Bodí, Mario Gutiérrez-Bedmar, Jorge Rodríguez-Capitán, Francisco Javier Pavón-Morón, Manuel Jiménez-Navarro

**Affiliations:** 1 Instituto de Investigación Biomédica de Málaga y Plataforma en Nanomedicina (IBIMA Plataforma BIONAND), 29010 Málaga, Spain; rjjimenez89@gmail.com (R.J.-L.); jlromerotrevejo@gmail.com (J.L.R.-T.); lfdezrmr@gmail.com (L.F.-R.); lauramartinchaves@gmail.com (L.M.-C.); miguel.romero@ibima.eu (M.R.-C.); molinaramos.ana@gmail.com (A.I.M.-R.); mjsquintero@gmail.com (M.J.S.-Q.); moramurri@gmail.com (M.M.); dottfrancescocosta@gmail.com (F.C.); bedmar@uma.es (M.G.-B.); mjimeneznavarro@gmail.com (M.J.-N.); 2 Emergency Department, Virgen de la Victoria University Hospital, Campus de Teatinos s/n, 29010 Málaga, Spain; 3 Department of Medicine and Dermatology, Faculty of Medicine, University of Málaga, Campus de Teatinos s/n, 29010 Málaga, Spain; 4 Ophthalmology Department, Virgen de la Victoria University Hospital, Campus de Teatinos s/n, 29010 Málaga, Spain; 5 Cardiology and Cardiovascular Surgery Department, Virgen de la Victoria University Hospital, Campus de Teatinos s/n, 29010 Málaga, Spain; 6 Centro de Investigación Biomédica en Red de Enfermedades Cardiovasculares (CIBERCV), Instituto de Salud Carlos III, 28029 Madrid, Spain; vicente.bodi@uv.es; 7 Centro de Investigación Biomédica en Red en Fisiopatología de Obesidad y Nutrición (CIBERObn), Instituto de Salud Carlos III, 28029 Madrid, Spain; 8 Department of Biomedical and Dental Sciences and Morphological and Functional Imaging, University of Messina, 98122 Messina, Italy; 9 Cardiology Department, Clinical University Hospital of Valencia, University of Valencia, Instituto de Investigación Sanitaria (INCLIVA), 46010 Valencia, Spain; 10 Department of Preventive Medicine and Public Health, Faculty of Medicine, University of Málaga, Campus de Teatinos s/n, 29010 Málaga, Spain

**Keywords:** cardiometabolic disorder, diabetes, coronary artery disease, tear, cytokine, ophthalmological parameter

## Abstract

**Background/Objectives**: Coronary artery disease (CAD) and type-2 diabetes mellitus (T2DM) are characterized by chronic low-grade inflammation. However, measuring cytokines typically involves invasive blood sampling, which can be problematic for CAD patients. This study aimed to assess ophthalmological parameters and tear cytokines in patients with CAD, comparing those with comorbid T2DM to those without to understand their inflammatory profiles. **Methods**: One hundred subjects with suspected chronic or acute CAD were initially included in this single-center cross-sectional study after clinical stabilization. Seventy-two patients with confirmed CAD were divided into two groups: 32 patients with T2DM and 40 patients without T2DM. A total of 144 eyes were examined, and tear fluid samples were collected to determine cytokine concentrations. Ophthalmological parameters and tear concentrations of cytokines were analyzed, controlling for age, sex, and other cardiovascular risk factors. **Results**: Patients with CAD and T2DM exhibited decreased ophthalmological parameters and increased cytokine concentrations in comparison to those without T2DM. Significant inverse correlations between ophthalmological parameters and cytokine concentrations were observed. Following adjustment, a full logistic regression model for distinguishing patients with CAD and comorbid T2DM included macular cube volume, mean macular thickness, interleukin (IL)-4, IL-5, IL-6, IL-8, IL-9, IL-13, granulocyte colony-stimulating factor (G-CSF), CCL3, CCL4, and CCL11/eotaxin-1, demonstrating excellent discriminatory power (Area Under the Curve = 0.95, 95% Confidence Interval = 0.91–0.99; *p* < 0.001). Subsequently, IL-5 (Odds Ratio = 1.68, 95% CI = 1.26–2.24; *p* < 0.001), G-CSF (OR = 1.06, 95% CI = 1.02–1.11; *p* < 0.01), and CCL11/eotaxin-1 (OR = 1.56, 95% CI = 1.19–2.05; *p* = 0.001) emerged as the most distinguishing variables in a reduced model (AUC = 0.89, 95% CI = 0.84–0.95; *p* < 0.001). **Conclusions**: Differences in ophthalmological variables, mainly in cytokine concentrations, suggest distinct pathophysiological mechanisms in patients with CAD based on the presence of T2DM. These findings demonstrate that the inflammatory profile can be readily detected through tear sample cytokines, proving valuable for establishing more accurate prognoses and monitoring in cardiometabolic disorders.

## 1. Introduction

Patients with cardiovascular diseases have a high comorbidity that complicates their therapeutic approach and significantly affects their prognosis. Among the most prevalent non-cardiovascular complications, type 2 diabetes mellitus (T2DM) is notable, affecting nearly 25% of patients with coronary artery disease (CAD) [1]. Additionally, T2DM is an independent risk factor for CAD, separate from traditional risk factors, such as hypertension, hyperlipidemia, and obesity. The presence of T2DM and insulin resistance is associated with a two- to threefold higher incidence of cardiovascular disease, myocardial infarction, and even death [2,3].

CAD and T2DM share pathophysiological bases through common alterations in gene expression and signaling systems involved in atherosclerosis and insulin resistance, including the inflammatory response [4]. Research on the role of the inflammatory system in the pathogenesis of T2DM has increased in recent years, revealing the abnormal secretion of proinflammatory mediators in insulin resistance and a reduction in anti-inflammatory mediators [5]. Both CAD and T2DM are now considered chronic low-grade inflammatory diseases with systemic alterations in cytokines, a large and diverse family of proteins or glycoproteins involved in modulating the immune system. Therefore, the detection of cytokines in biological fluids, such as blood and cerebrospinal fluid, represents a fundamental tool for the early diagnosis or monitoring of conditions or abnormalities associated with inflammatory alterations, including CAD and T2DM [6]. Interestingly, recent evidence suggests that distinct inflammatory cytokine profiles associated with cardiometabolic conditions could help discriminate between patients with varying risks [7].

Inflammation drives the development of CAD, which encompasses a spectrum of diseases primarily related to the coronary arteries, including atherosclerosis, thrombosis, acute myocardial, and ischemia-reperfusion injury, and alterations in circulating cytokines have been extensively implicated in these processes. Specifically, elevated concentrations of proinflammatory cytokines, such as interleukin-1 beta (IL-1β), tumor necrosis factor-alpha (TNF-α), and interferon-gamma (IFN-γ), are associated with the increased risks of acute coronary syndrome [8]. Additionally, a recent study demonstrated that the combination of serum cytokines and clinical risk factors, including diabetes, may help identify patients with more severe coronary artery lesions from those with suspected CAD but not acute myocardial infarction [9].

The role of inflammation in diabetes remains under investigation, with a relationship established between the inflammatory response and the development of microvascular diseases, such as diabetic nephropathy [10]. Indeed, studies have shown a connection between acute-phase inflammatory markers and nephropathy, demonstrating the role of inflammation in the pathogenesis of diabetic glomerulopathy [11]. Further research is necessary to better characterize the causal relationship of proinflammatory cytokines, hypoadiponectinemia, and the positive regulation of anti-inflammatory proteins before the onset of T2DM [12].

Despite efforts to characterize serum cytokines to stratify patients with CAD based on disease severity [13], the presence of different cardiovascular risk factors, such as T2DM, also significantly impacts these mediators, making it difficult to draw solid conclusions and identify reliable biomarkers. This has driven the search for new methodological strategies and a more comprehensive clinical characterization of study participants to determine specific inflammatory profiles.

Unlike blood collection, tear collection for cytokine analysis is a novel and promising method in biomedical research due to its non-invasive nature, which increases the availability of biomaterial, reduces the risks of potential complications, and decreases concomitant costs [14]. Consequently, the use of tear cytokine determinations in combination with ophthalmological parameters has increased in recent years for various pathologies, primarily ocular diseases [15,16,17]. Although very few studies focused on cardiovascular diseases have explored the use of tear cytokines, a recent study by our group found an association between CAD and certain cytokines in tears, with an observed increase in granulocyte colony-stimulating factor (G-CSF) alongside the presence of CAD [18]. Regarding T2DM, most studies using tear samples have focused on ocular diseases related to its severity, such as diabetic retinopathy, with significant inconsistencies observed in tear concentrations of cytokines like TNF-α and IL-8 [19,20].

Given the limited research on cardiovascular diseases using ophthalmological variables, identifying specific inflammatory and ophthalmological profiles may prove valuable for effectively managing and evaluating risk in patients with CAD and prevalent cardiovascular risk factors using non-invasive procedures. Therefore, the primary objective of this study in patients with CAD was to determine relevant tear cytokines in combination with ophthalmological parameters and to establish distinct inflammatory profiles based on the coexistence or absence of T2DM. This study accounted for other cardiovascular risk factors, including age and sex, while excluding patients with ocular diseases. Alterations in these ophthalmological variables may also suggest potential biomarkers related to the severity and risk of both cardiometabolic disorders, warranting further investigation.

## 2. Materials and Methods

### 2.1. Study Design and Participants

This single-center cross-sectional study was conducted in the Cardiology and Ophthalmology departments of the Virgen de la Victoria University Hospital (Málaga, Spain). To prevent selection bias, the study included consecutive patients who sought assistance at the hospital over a six-month period (February to July 2022) due to suspected chronic or acute CAD was based on clinical presentation.

At the start of the study, after clinical stabilization, 125 patients with suspected CAD were initially recruited due to the presence of symptoms (e.g., chest pain, fatigue, dizziness) and classical cardiovascular risk factors consistent with CAD. After reviewing the electronic medical records and applying the eligibility criteria, 25 patients were excluded from the study. Thus, 100 patients with suspected CAD met the eligibility criteria and were clinically assessed through coronary examination (i.e., coronary angiography and/or computed tomography of the coronary arteries). These patients were then categorized into those with and without a CAD diagnosis. Subsequently, 72 patients with confirmed CAD were grouped into two groups based on the coexistence of T2DM: the non-T2DM (40 patients) and T2DM (32 patients) groups. Some of these patients with suspected CAD had previously participated in a prior investigation [18].

### 2.2. Eligibility Criteria

Participation was voluntary, and all patients who fulfilled the eligibility criteria signed their informed consents and were examined through clinical assessments (cardiac and ophthalmological assessments) and medical records. Exclusion criteria included the presence of any disease that reduced life expectancy to less than one year, an estimated glomerular filtration rate (eGFR) of less than 30 mL/min/1.73 m^2^ (i.e., severe chronic kidney disease at stages 4 and 5), active cancer, and chronic inflammatory or infectious diseases associated with sustained elevation of circulating cytokines. As exclusive ophthalmological diseases, the presence of amblyopia in either eye, having undergone retinal photocoagulation in the past, or suffering from any type of retinal disease, including diabetic or hypertensive retinopathy, diabetic macular edema, vein occlusions, retinal dystrophies, epiretinal membranes, vitreomacular traction, age-related macular degeneration, or central serous chorioretinopathy, were also included.

### 2.3. Coronary Assessment and Clinical Characteristics

#### 2.3.1. Coronary Angiography and Computed Tomography

To evaluate the presence of coronary lesions and the severity of CAD, patients underwent coronary assessments. Specifically, these assessments were scheduled for patients with chronic CAD (i.e., stable angina) and were conducted within the first 48 h for those hospitalized with acute CAD [i.e., ST-segment elevation myocardial infarction (STEMI) and non-ST-segment elevation myocardial infarction (NSTEMI)]. Coronary angiography was the primary diagnostic tool used in all cases; however, computed tomography was also utilized for patients with suspected chronic CAD, either in conjunction with or independently of coronary angiography.

Patients with CAD had ≥70% stenosis in any major epicardial artery (right coronary artery, left anterior descending, or circumflex marginal branch) or >50% in the left main coronary artery when coronary angiography was performed. Regarding computed tomography of the coronary arteries, patients with ≥50% stenosis in any coronary artery were diagnosed patients with CAD.

#### 2.3.2. Baseline Clinical Characteristics

Demographic and clinical data were collected for each patient through electronic medical records, medical examinations during hospitalization, and coronary assessment. Consequently, classical cardiovascular risk factors [e.g., sex, age, smoking habit, T2DM, hypertension, hypercholesterolemia, obesity, chronic obstructive pulmonary disease (COPD), and chronic kidney disease (excluding severe stages 4 and 5)] and prior cardiovascular diseases were documented.

Regarding CAD-related variables, clinical information was obtained on the type of CAD, its severity based on coronary lesions, biochemical parameters, and treatment type [e.g., percutaneous coronary intervention (PCI) and coronary artery bypass graft surgery (CABG)].

### 2.4. Ophthalmological Assessment and Collection of Tear Samples

Ophthalmological assessment was conducted 24 h before hospital discharge or within the first 7 days following the diagnosis of chronic CAD in outpatient settings. This timing was selected to ensure clinical stability across all patients. Various ophthalmological parameters were measured, and tear samples were collected at the conclusion of these assessments.

All ophthalmological examinations were performed by the same ophthalmologist at a consistent time of day (between 3:00 p.m. and 6:00 p.m.).

#### 2.4.1. Ophthalmological Parameter Determination

Several parameters were measured during the ophthalmological assessments, such as best-corrected visual acuity (BCVA) using numerical optometric charts, intraocular pressure (IOP) using a Perkins Mk3 tonometer (Haag-Streit, Essex, UK), Schirmer’s test using Schirmer-Plus^®^ strips (GECIS, Neung-sur-Beuvron, France), central corneal thickness (TCCT and PCCT) using an Orbscan^®^ IIz topographer (Bausch and Lomb, Vaughan, ON, Canada) and an OcuScan^®^ RxP ultrasound pachymeter (Alcon Laboratories, Fort Worth, TX, USA), axial length using an IOLMaster^®^ 500 optical biometer (Carl Zeiss Meditec AG, Jena, Germany), and several variables obtained from optical coherence tomography (OCT) using the Cirrus™ high-definition (HD)-OCT system (Carl Zeiss Meditec AG, Jena, Germany), such as central macular thickness (CMT), macular cube volume (MCV), mean macular thickness (MMT), retinal nerve fiber layer thickness (RNFLT), ganglion cell layer thickness (GCLT), and ganglion cell layer minimum thickness (GCLMT). Finally, the choroidal thickness was manually calculated using the device’s ruler from the images, defined as the distance between the external hyper-reflective band of the retinal pigment epithelium and the internal hyper-reflective band of the sclera [8]. Ophthalmological parameters were measured in the 144 eyes of 72 patients with CAD (n = 144, total sample; n = 40, non-T2DM group; and n = 32, T2DM group).

#### 2.4.2. Collection of Tear Samples

In addition to the aforementioned ophthalmological parameters, tear samples were collected from each patient using a paper strip that was placed in the inferior fornix of each eye without prior topical anesthesia. The strip was left in place for 5 min and then immediately frozen at −80 °C until analysis. Samples with less than 6 mm of wetness on the strip after the 5-min collection period were excluded from analysis.

### 2.5. Processing of Tear Samples and Determination of Inflammatory Mediators

To elute proteins, each paper containing a sample of tear fluid was fragmented into small pieces, which were subsequently immersed in 100 μL of phosphate-buffered saline (PBS) containing 0.3% Tween^®^ 20, 0.5% bovine serum albumin (BSA), and a protease inhibitor. The mixture was then incubated overnight at 4 °C, after which the supernatant was collected. The quantification of total protein content in each sample was performed using a NanoDropTM One spectrophotometer (Thermo Fischer Scientific, Waltham, MA, USA) by measuring the absorbance at 280 nm.

The determination of cytokine concentrations was performed following the instructions of the Bio-Plex Pro^TM^ Human Cytokine 27-plex Assay kit (#M500KCAF0Y, Bio-Rad Laboratories, Hercules, CA, USA). A total of 27 inflammatory mediators were analyzed: interleukin (IL)-1β, IL-1ra, IL-2, IL-4, IL-5, IL-6, IL-7, IL-8, IL-9, IL-10, IL-12p70, IL-13, IL-15, IL-17A, tumor necrosis factor (TNF)-α, vascular endothelial growth factor (VEGF), fibroblast growth factor (FGF)-2, granulocyte colony-stimulating factor (G-CSF), granulocyte-macrophage colony-stimulating factor (GM-CSF), interferon (IFN)-γ, platelet-derived growth factor (PDGF)-BB, eotaxin-1 (CCL11), monocyte chemoattractant protein (MCP)-1 (CCL2), macrophage inflammatory protein (MIP)-1α (CCL3), MIP-1β (CCL4), regulated on activation normal T cell expressed and secreted (RANTES) (CCL5), and IFN-γ-induced protein 10 (IP-10) (CXCL10). The 96-well plates were measured using a Bio-Plex MAGPIX™ reader and Bio-Plex Manager™ MP software version 6.2 (Luminex, Austin, TX, USA) in the Proteomics Unit at the Central Research Support Services of the University of Málaga.

All samples were run in duplicate to enhance the reliability and accuracy of measurements and to minimize measurement bias. For samples with an optical density (OD) lower than the limit of detection in the multiplex assay but higher than the background (zero values), the assignment of concentrations was arranged from the sample with the lowest OD, which was assigned with half of the minimum concentration that could be interpolated in the standard curves [21]. The intra-assay coefficient of variability (CV) was less than 8%, and the inter-assay CV was less than 10%. Tear concentrations of these inflammatory mediators were measured in pg/mL or ng/mL for the 144 eyes of 72 patients with CAD (n = 144, total sample; n = 40, non-T2DM group; and n = 32, T2DM group).

### 2.6. Statistical Analysis

In the planning phase of our study, we conducted a sample size calculation to guarantee the statistical power required for robust and meaningful results, particularly in relation to tear cytokine concentrations. Our objective was to identify a clinically significant difference with 80% power at a significance level of 0.05, drawing upon insights from prior studies focused on tear cytokine levels [18,20]. Moreover, to account for potential dropouts and the intricacies of our study design, which involved recruiting individuals with non-confirmed acute CAD, we made adjustments to the sample size. This recalibration considered the statistical methods specific to our study objectives, ensuring not only the statistical validity of our sample size but also its adequacy for deriving valid conclusions.

Data were expressed as the number and percentage of events [n (%)], mean and standard deviation (mean ± SD), or median and interquartile range [median (IQR, 25–75%)] depending on the type of variable and distribution.

The statistical significance of differences in categorical variables was assessed using Fisher’s exact test, while the significance of differences in continuous variables was calculated using either the Mann–Whitney U test for non-normally distributed variables or the Student *t*-test for normally distributed variables. To control for the false discovery rate (FDR) resulting from multiple comparisons between the T2DM and non-T2DM groups, we applied the Benjamini–Hochberg procedure.

A multiple correlation analysis was performed between the ophthalmological parameters and tear cytokine concentrations using the Spearman correlation coefficient (rho).

Subsequently, an analysis of covariance (ANCOVA) (*F* statistic) was performed to assess the association between the presence of comorbid T2DM (the non-T2DM and T2DM groups) and both ophthalmological parameters and tear inflammatory cytokines. This analysis involved adjusting for other independent variables and covariates (i.e., age, sex, and prevalent classical cardiovascular risk factors) to prevent biased associations. Raw data of cytokine concentrations were log10-transformed to approximate a normal distribution and to ensure statistical assumptions of the ANCOVA. The estimated marginal means and 95% confidence intervals (95% CI) of the log10-transformed cytokine concentrations were back-transformed and represented.

Binary logistic regression analyses to differentiate patients with T2DM from those without in the context of CAD were performed, using as explanatory variables both ophthalmological parameters and tear concentrations of inflammatory cytokines. The explanatory variables were rescaled as necessary to facilitate the interpretation of their regression coefficients and odds ratios (OR). The resulting model(s) were analyzed using both the Hosmer–Lemeshow test for the goodness of fit and the receiver operating characteristic (ROC) curve for the discriminatory power.

All statistical analyses were performed using the GraphPad Prism version 5.04 (GraphPad Software, San Diego, CA, USA), and IBM SPSS Statistics version 22 (IBM, Armonk, NY, USA). *p*-values and adjusted *p*-values (*q*-values) less than 0.05 were considered statistically significant.

## 3. Results

### 3.1. Clinical Characteristics of Patients with CAD

After clinical stabilization, 100 patients with suspected chronic or acute CAD met the eligibility criteria and were divided into patients with CAD diagnosis and patients without CAD diagnosis (Table S1). The patients with suspected CAD were predominantly men (27% women) with a mean age of 60 years. Seventy-two patients were confirmed to have CAD after coronary assessments; however, there were no significant differences in the prevalence of classical cardiovascular risk factors between those with and without CAD. This outcome was expected, as the indication for coronary angiography at our center is based on estimating cardiovascular risk, which is primarily determined by the presence of these risk factors.

Patients diagnosed with CAD (n = 72) were categorized based on the presence or absence of T2DM, revealing a high prevalence of classical cardiovascular risk factors (Table 1). Specifically, 50% of the patients had hypertension, 44% were smokers, 42% were obese, and 35% had hypercholesterolemia. Although patients with T2DM exhibited a higher prevalence of comorbidities and prior cardiovascular diseases, such as hypercholesterolemia, obesity, and hypertension, compared to those without T2DM, the differences were not statistically significant. Regarding CAD-related characteristics, no significant differences were observed between patients with and without T2DM across the various variables in our sample. The majority of patients had NSTEMI (42%) and multi-vessel disease (46%). The preferred treatments were PCI or CABG, utilized in 68% of patients with CAD.

### 3.2. Ophthalmological Parameters and Tear Cytokine Concentrations in Patients with CAD Based on T2DM

Patients with CAD were grouped based on the presence or absence of T2DM, and their eyes were assessed by collecting ophthalmological parameters and tear samples. There were no missing values.

#### 3.2.1. Ophthalmological Parameters

As depicted in Table 2, numerous ophthalmological parameters were assessed, and some differences were observed in patients with CAD and comorbid T2DM compared to those patients with CAD but not T2DM (non-T2DM group).

Although there was a decrease in the Schirmer’s test, BCVA, and GCLMT among patients with comorbid T2DM, these differences were not statistically significant. However, patients with comorbid T2DM exhibited significantly lower MCV (*q* < 0.01), MMT (*q* < 0.01), RNFLT (*q* < 0.05), and GCLT (*q* < 0.05) compared to patients without T2DM after adjusting *p*-values for multiple comparisons.

#### 3.2.2. Tear Cytokine Concentrations

Some analytes could not be quantified in the tear samples due to concentrations falling below the limit of detection: IL-2, IL-10, IL-12p70, IL-15, and VEGF.

Overall, the tear concentrations of inflammatory mediators were higher in patients with CAD and comorbid T2DM than in patients without T2DM (Table 3). Specifically, IL1-ra, IL-4, IL-5, IL-6, IL-8, IL-9, IL-13, G-CSF, GM-CSF, IFN-γ, CCL3/MIP-1α, CCL4/MIP-1β, and CCL11/eotaxin-1 concentrations were found to be significantly increased (*q* < 0.05) after adjusting *p*-values for multiple comparisons. Conversely, the concentrations of relevant proinflammatory cytokines, such as IL-1β and TNF-α, exhibited no significant differences between the two groups of patients with CAD.

### 3.3. Correlation between Significant Ophthalmological Parameters and Tear Cytokine Concentrations in Patients with CAD

We investigated the potential association of those variables that were found to be statistically different in both groups of patients with CAD.

**Figure 1:**
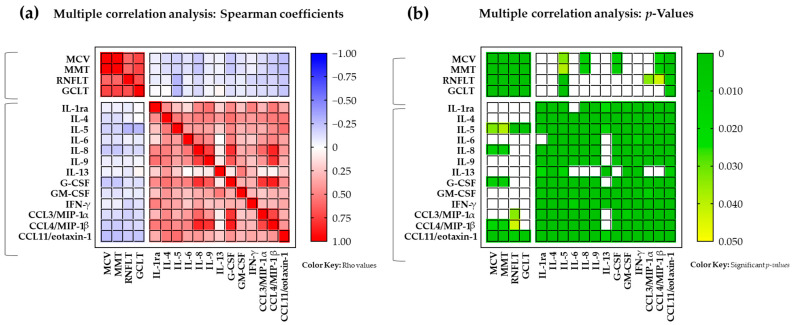
Multiple correlation analysis between ophthalmological parameters and tear inflammatory cytokines from patients with CAD diagnosis. The variables were analyzed using Spearman’s correlation coefficients: (**a**) Rho values were color-coded using a key ranging from blue (rho = −1.0) to red (rho = +1.0); and (**b**) significant *p*-values were also color-coded using a key ranging from green (*p* < 0.001) to yellow (*p* < 0.05). Ophthalmological parameters were measured in the 144 eyes of 72 patients with CAD. Tear cytokine concentrations were measured in duplicate for each of the 144 eyes. Single determinations of ophthalmological parameters and the mean concentrations of cytokines were used in the multiple correlation analysis (n = 144).

Thus, there were inverse correlations between ophthalmological parameters (i.e., MCV, MMT, RNFLT, and GCLT) and the majority of tear cytokine concentrations (i.e., IL1-ra, IL-4, IL-5, IL-6, IL-8, IL-9, G-CSF, IFN-γ, CCL3/MIP-1α, CCL4/MIP-1β, and CCL11/eotaxin-1) (Figure 1).

These associations were statistically significant between ophthalmological parameters and the concentrations of certain inflammatory mediators (Figure 1a,b): IL-5 [MCV (rho = −0.18, *p* = 0.032); MMT (rho = −0.18, *p* = 0.039); RNFTL (rho = −0.29, *p* < 0.001); and GCLT (rho = −0.28, *p* = 0.001)], IL-8 [MCV (rho = −0.21, *p* = 0.011); and MMT (rho = −0.22, *p* = 0.009)], G-CSF [MCV (rho = −0.22, *p* = 0.010); and MMT (rho = −0.22, *p* = 0.008)], CCL3/MIP-1α [RNFLT (rho = −0.18, *p* = 0.033)], CCL4/MIP-1β [MCV (rho = −0.20, *p* = 0.016); MMT (rho = −0.21, *p* = 0.013); and RNFLT (rho = −0.17, *p* = 0.043)], and CCL11/eotaxin-1 [MCV (rho = −0.26, *p* = 0.002); MMT (rho = −0.25, *p* = 0.003); RNFLT (rho = −0.22, *p* = 0.010); and GCLT (rho = −0.23, *p* = 0.006)].

### 3.4. Influence of Other Age, Sex, and Classical Cardiovascular Risk Factors on Significant Ophthalmological Parameters and Tear Cytokine Concentrations in Patients with CAD Based on T2DM

Considering the potential influence of sex, age, and other cardiovascular risk factors with higher prevalence (i.e., hypertension, hypercholesterolemia, obesity, and smoking habit) on the observed differences between patient groups with CAD based on comorbid T2DM, ANCOVAs were conducted for those statistically selected ophthalmological parameters and tear cytokine concentrations. COPD and chronic kidney disease were excluded from these analyses due to their low prevalence in both groups (less than 10%).

In order to ensure that statistical assumptions for parametric testing were met, logarithmic transformation was applied to the raw cytokine concentration data (pg/mL). The estimated marginal means were depicted subsequent to back-transformation in instances where there were significant differences between patients with CAD and comorbid T2DM and those patients without T2DM.

#### 3.4.1. Effects of Comorbid T2DM and Other Cardiovascular Risk Factors on Significant Ophthalmological Parameters

**Figure 2:**
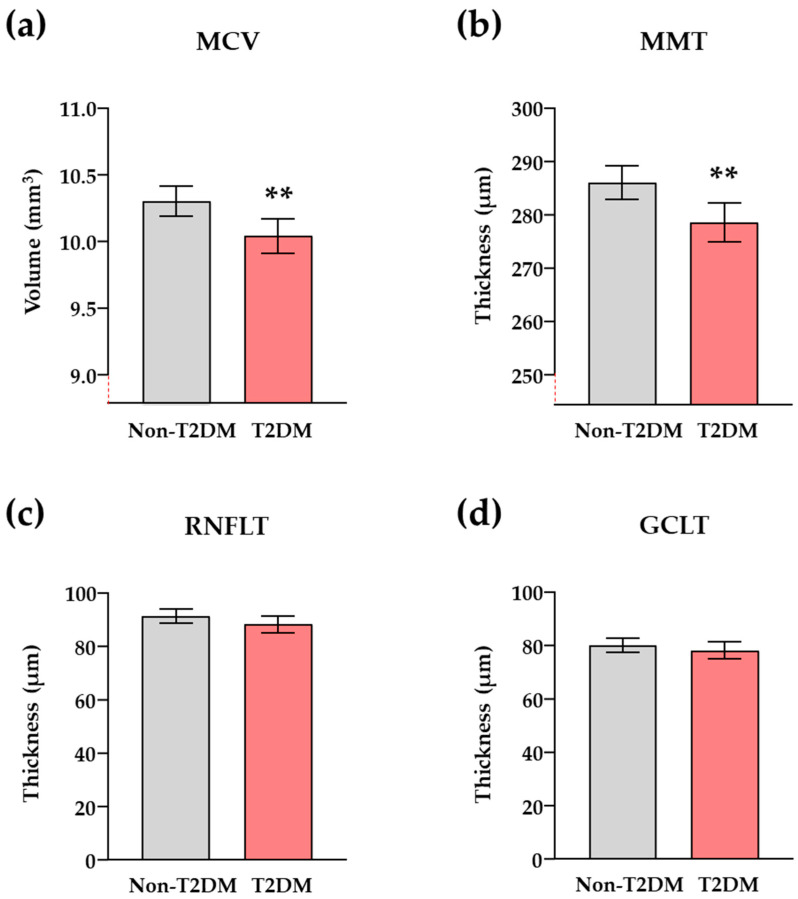
Ophthalmological parameters in patients with CAD based on the coexistence of T2DM after adjusting for other cardiovascular risk factors. (**a**) MCV; (**b**) MMT; (**c**) RNFLT; and (**d**) GCLT. Bars represent the estimated marginal means and 95% CI of dependent variables after ANCOVA with comorbid T2DM as main factor and hypertension, hypercholesterolemia, obesity, smoking habit, age, and sex as independent variables. (**) denotes significant differences with *p* < 0.01 compared to the non-T2DM group. Ophthalmological parameters were measured in the 144 eyes of 72 patients with CAD (n = 144, total sample; n = 80, non-T2DM group; and n = 64, T2DM group).

Regarding ophthalmological parameters in Figure 2 (i.e., MCV, MMT, RNFLT, and GCLT), patients with CAD and comorbid T2DM had significantly lower MCV (*F*_1, 132_ = 7.62, *p* = 0.007; Figure 2a) and MMT (*F*_1, 132_ = 8.11, *p* = 0.005; Figure 2b) compared to patients without T2DM. However, no differences were observed in RFNLT and GCLT between both patient groups after adjusting for age, sex, and other cardiovascular risk factors (Figure 2c,d).

Notably, only obesity showed a significant effect on all these ophthalmological parameters (*p* < 0.01), unlike other risk factors.

#### 3.4.2. Effects of Comorbid T2DM and Other Cardiovascular Risk Factors on Significant Cytokine Concentrations

**Figure 3:**
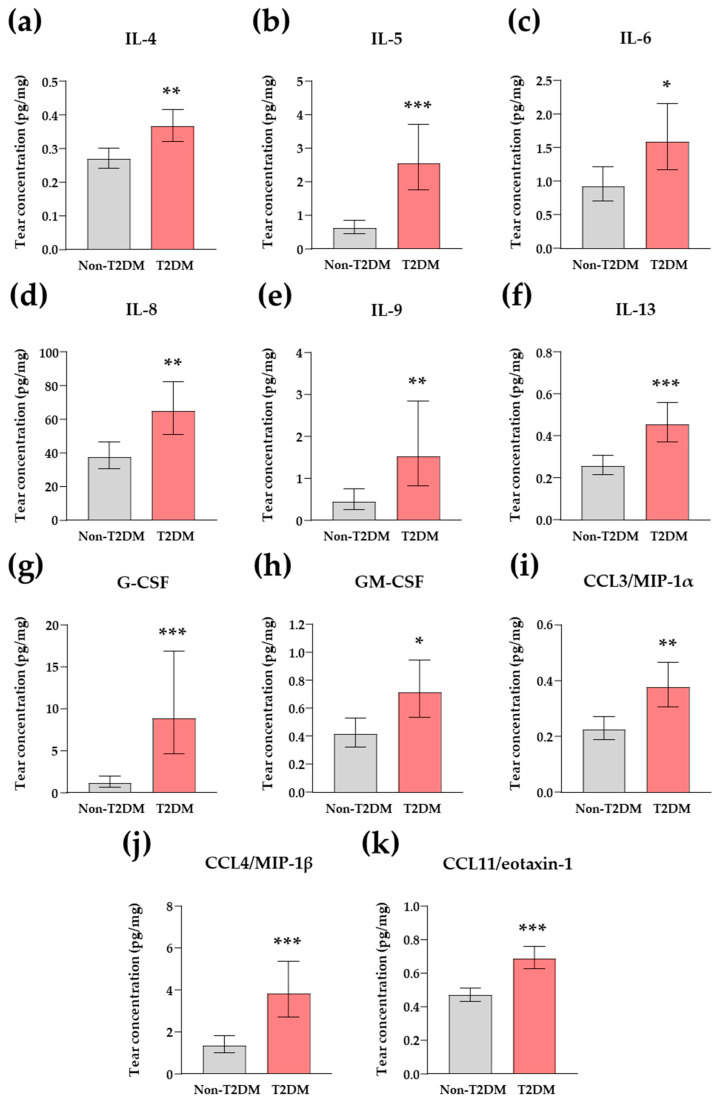
Tear cytokine concentrations in patients with CAD based on the coexistence of T2DM after adjusting for other cardiovascular risk factors. (**a**) IL-4; (**b**) IL-5; (**c**) IL-6; (**d**) IL-8; (**e**) IL-9; (**f**) IL-13; (**g**) G-CSF; (**h**) GM-CSF; (**i**) CCL3/MIP-1α; (**j**) CCL4/MIP-1β; and (**k**) CCL11/eotaxin-1. Bars represent the estimated marginal means and 95% CI of dependent variables after ANCOVA with comorbid T2DM as main factor and hypertension, hypercholesterolemia, obesity, smoking habit, age, and sex as independent variables. Raw cytokine concentrations were logarithmically transformed for analysis and back-transformed for representation. (*), (**), and (***) denote significant differences with *p* < 0.05, *p* < 0.01, and *p* < 0.001 compared to the non-T2DM group. Raw cytokine concentrations were measured in duplicate for each of the 144 eyes of 72 patients with CAD (n = 144, total sample; n = 80, non-T2DM group; and n = 64, T2DM group).

The ANCOVA results revealed that IL-1ra and IFN-γ concentrations were not significantly influenced by T2DM in the presence of other cardiovascular risk factors. However, as shown in Figure 3, patients with CAD and comorbid T2DM had significantly higher concentrations of IL-4 (*F*_1, 136_ = 10.54, *p* = 0.001; Figure 3a), IL-5 (*F*_1, 136_ = 26.21, *p* < 0.001; Figure 3b), IL-6 (*F*_1, 136_ = 5.75, *p* = 0.018; Figure 3c), IL-8 (*F*_1, 136_ = 9.52, *p* = 0.002; Figure 3d), IL-9 (*F*_1, 136_ = 7.54, *p* = 0.007; Figure 3e), IL-13 (*F*_1, 136_ = 15.09, *p* < 0.001; Figure 3f), G-CSF (*F*_1, 136_ = 19.06, *p* < 0.001; Figure 3g), GM-CSF (*F*_1, 136_ = 6.65, *p* = 0.011; Figure 3h), CCL3/MIP-1α (*F*_1, 136_ = 10.81, *p* = 0.001; Figure 3i), CCL4/MIP-1β (*F*_1, 136_ = 16.85, *p* < 0.001; Figure 3j), and CCL11/eotaxin-1 (*F*_1, 136_ = 28.48, *p* < 0.001; Figure 3k) than patients without T2DM.

While there were no significant effects of sex, hypertension, and obesity on these inflammatory cytokines, other independent factors exhibited significant effects: age on IL-8, IL-9, IL-13, G-CSF, and CCL4/MIP-1β concentrations; hypercholesterolemia on CCL3/MIP-1α concentrations; and smoking habit on IFN-γ concentrations.

### 3.5. Logistic Regression Models to Differentiate Patients with CAD Based on the Coexistence or Not of T2DM

To identify potential discriminatory variables, multivariable binary logistic regression analyses were conducted using ophthalmological parameters and raw cytokine concentrations that exhibited significant associations with the presence of comorbid T2DM while accounting for age, sex, and other classical cardiovascular risk factors.

#### 3.5.1. Full Logistic Regression Model

A full binary logistic regression model was constructed using MCV, MMT, and tear concentrations of IL-4, IL-5, IL-6, IL-8, IL-9, IL-13, G-CSF, GM-CSF, CCL3/MIP-1α, CCL4/MIP-1β, and CCL11/eotaxin-1 (Table 4). The model identified MCV (mm^3^ rescaled by multiplying by 100: OR = 1.41; 95% CI = 1.07–1.85; *p* < 0.05), MMT (OR = 0.29; 95% CI = 0.11–0.77; *p* < 0.05), IL-4 (pg/mL rescaled by multiplying by 10: OR = 0.43; 95% CI = 0.20–0.90; *p* < 0.05), IL-5 (OR = 2.09; 95% CI = 1.43–3.05; *p* < 0.001), IL-13 (pg/mL rescaled by multiplying by 10: OR = 1.47; 95% CI = 1.09–1.98; *p* < 0.01), G-CSF (OR = 1.44; 95% CI = 1.18–1.75; *p* < 0.001), CCL4/MIP-1α (pg/mL rescaled by multiplying by 10: OR = 0.41; 95% CI = 0.20–0.85; *p* < 0.05), and CCL-11/eotaxin-1 (pg/mL rescaled by multiplying by 10: OR = 2.68; 95% CI = 1.61–4.47; *p* < 0.001) as distinguishing variables for comorbid T2DM in patients with CAD.

**Figure 4:**
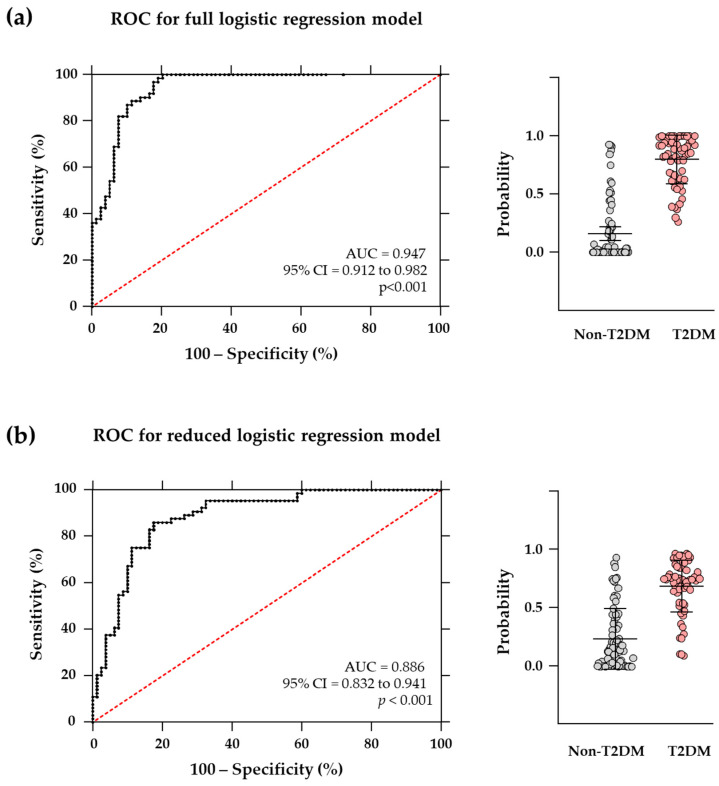
ROC analysis for binary logistic regression models to distinguish patients with CAD and comorbid T2DM from those patients with CAD but not T2DM. (**a**) ROC analysis for the full logistic regression model using significant ophthalmological parameters and log_10_-transformed cytokine concentrations; and (**b**) ROC analysis for the reduced logistic regression model using log_10_ IL-5, log_10_ G-CSF, and log_10_ CCL11/eotaxin-1. Probabilities obtained from each binary logistic regression model (n = 144, total sample; n = 80, non-T2DM group; and n = 64, T2DM group) were used for both ROC analyses. Red dashed lines represent a perfectly random classification.

The ROC analysis demonstrated a high discriminatory power of the model (AUC = 0.950, 95% CI = 0.914–0.985; *p* < 0.001). An optimal cut-off value of 0.487 was chosen, resulting in 93.4% sensitivity and 91.1% specificity (Figure 4a).

#### 3.5.2. Reduced Logistic Regression Model

Subsequently, the model resulting from a strict backward Wald stepwise method (i.e., probability for stepwise: entry = 0.05 and removal = 0.01) included only three explanatory variables: IL-5 (OR = 1.68; 95% CI = 1.26–2.24; *p* < 0.001), G-CSF (OR = 1.06; 95% CI = 1.02–1.11; *p* < 0.01), and CCL11/eotaxin-1 (OR = 1.56; 95% CI = 1.19–2.05; *p* = 0.001) concentrations (Table 4).

This well-fitting model maintained a high discriminatory power (AUC = 0.891, 95% CI = 0.837–0.945; *p* < 0.001). An optimal cut-off value for the reduced model was 0.378 (85.7% sensitivity and 80.1% specificity) (Figure 4b).

Additionally, individual logistic regression and ROC analyses confirmed IL-5, G-CSF, and CCL11/eotaxin-1 concentrations as the most distinguishing variables for comorbid T2DM in patients with CAD (Table S2).

## 4. Discussion

Regular ophthalmological monitoring is crucial for diabetic patients to prevent future complications associated with microvascular damage, such as diabetic retinopathy, which are often preceded by an inflammatory state. Additionally, it serves as an indicator of disease progression. Thus, periodic fundus examinations and ophthalmological analyses are performed as screening and prognostic tools [22]. However, these ophthalmological assessments may also prove to be immensely useful in the context of CAD, once the determination of inflammatory mediators in tear samples can be implemented. This study represents the first attempt to evaluate the utility of ophthalmological variables, mainly derived from tear cytokines, in patients with cardiometabolic disorder.

In summary, the main findings of this study are as follows: (1) Patients with diagnosed CAD and comorbid T2DM exhibited significantly lower MCV, MMT, RNFLT, and GCLT in their eyes than patients without T2DM after adjusting for multiple comparisons; (2) Patients with CAD and comorbid T2DM had significantly higher tear concentrations of IL-1ra, IL-4, IL-5, IL-6, IL-8, IL-9, IL-13, G-CSF, GM-CSF, IFN-γ, CCL3/MIP-1α, CCL4/MIP-1β, and CCL11/eotaxin-1 than patients without T2DM after adjusting for multiple comparisons; (3) There were inverse correlations between ophthalmological parameters and inflammatory mediators; and (4) Logistic regression models were developed to discriminate comorbid T2DM among patients with CAD, incorporating specifically selected ophthalmological parameters and tear cytokine concentrations based on their statistical significance while controlling for the influence of age, sex, and other prevalent classical cardiovascular risk factors (i.e., hypertension, hypercholesterolemia, obesity, and smoking habits). Among the ophthalmological variables assessed, IL-5, G-CSF, and CCL11/eotaxin-1 concentrations were identified as the most distinguishing variables to discriminate patients with CAD and comorbid T2DM from those without T2DM. Therefore, distinctive inflammatory profiles in patients with CAD can be identified through tear cytokines using a rapid, safe, and non-invasive technique. These differences may indicate different pathophysiological inflammatory mechanisms in CAD, depending on the presence or absence of metabolic diseases, such as T2DM. Considering the straightforward identification of these specific inflammatory patterns in each patient group, tear cytokine monitoring could be valuable in improving our understanding of the inflammatory status and prognosis in these patients.

Patients with CAD exhibited certain changes in ophthalmological parameters when examined based on the presence or not of comorbid T2DM. Specifically, we found differences in MCV, MMT, RFNLT, and GCLT measurements with significantly lower values in patients with comorbid T2DM. Many ocular pathologies are associated with T2DM (e.g., diabetic retinopathy), and thus, although many of them have been excluded in this study as eligibility criteria, we cannot rule out that these findings may be related to a preclinical state of such pathologies. In agreement with this suggestion, several studies have reported a decrease in macular thickness in patients with T2DM and minimal diabetic retinopathy and even in patients with prediabetes compared to controls [23,24]. Some authors have linked this macular decrease (e.g., MCV and MMT) with a neurodegeneration in early stages, which may explain the loss of RNFLT and GCLT also observed in patients with T2DM who have minimal or no diabetic retinopathy in accordance with our findings [25,26]. Furthermore, we cannot disregard the impact of other cardiovascular risk factors besides T2DM on these ophthalmological parameters among patients with CAD. Actually, the statistical analysis of our data also revealed a significant main effect of obesity on MCV, MMT, RNFLT, and GCLT, which is consistent with numerous studies that have shown a clear association between these parameters and obesity [27,28]. The impact of body mass on ophthalmological parameters could diminish their specificity or ability to differentiate patients with comorbid T2DM from those without T2DM in the logistic models, unlike tear inflammatory cytokines.

Cytokines are a broad family of small proteins with pleiotropic effects that act on cells to modulate inflammatory and immunological responses. They play a crucial role in the pathophysiology of a variety of cardiometabolic diseases and have been assessed as inflammatory biomarkers [29,30]. Therefore, the measurement of cytokines in bodily fluids is a fundamental approach for early detection of CAD and T2DM due to their underlying inflammatory mechanisms. While serum and plasma samples have been the preferred fluids for analysis in the majority of studies, other biological fluids have been used depending on the target organs affected by the disease. For example, cerebrospinal fluid is used to evaluate cognitive damage in patients with Alzheimer’s disease, and ascitic fluid to assess bacterial peritonitis in patients [31,32]. Our study focuses primarily on ocular involvement associated with complications of T2DM and atherosclerosis in patients with CAD. Therefore, we have focused on obtaining tear samples for cytokine determination as a novel and non-invasive technique in the field of cardiovascular diseases. Recently, we have found differences in certain cytokines within tear samples obtained from patients with suspected CAD, indicating a potential prognostic significance [18]. However, no overall increase in inflammation was observed in association with the CAD diagnosis. On the contrary, the current findings provide clear evidence that the occurrence of T2DM in patients with CAD was linked to an increased inflammatory state, which was characterized by increased concentrations of inflammatory cytokines from tear samples given its systemic nature. Therefore, there were significant increases in several inflammatory mediators, specifically in IL-1ra, IL-4, IL-5, IL-6, IL-8, IL-9, IL-13, G-CSF, GM-CSF, IFN-γ, CCL3/MIP-1α, CCL4/MIP-1β, and CCL11/eotaxin-1, though IL-1ra and IFN-γ concentrations were excluded after adjusting for age, sex, and other classical cardiovascular risk factors. The construction of a logistic regression model with these tear cytokines to distinguish patients with CAD and comorbid T2DM from those without T2DM revealed IL-5, G-CSF, and CCL11/eotaxin-1 concentrations as promising inflammatory biomarkers associated with comorbid T2DM in the tear fluid of these patients. Noticeably, despite the absence of diabetic retinopathy, IL-5, G-CSF, and CCL11/eotaxin-1 concentrations demonstrated a significant inverse correlation with MCV and MMT. While we cannot establish causality for the association between macular parameters and tear cytokine concentrations, a previous study has suggested that changes in inflammatory cytokines in the aqueous humor, including elevated CCL11/eotaxin-1, may contribute to decreased macular thickness following intravitreal treatment for diabetic macular edema [33].

IL-5 is primarily produced by type 2 helper T lymphocytes (Th2) but can also be produced by mast cells and, to a lesser extent, by eosinophils. The number of studies investigating IL-5 levels in the blood of patients with diabetes, including prediabetes and metabolic syndrome, is limited and the results are contradictory. Therefore, the concentrations of IL-5 have been shown to fluctuate depending on the clinical context, with both elevated and reduced levels being reported [34,35]. Regarding CAD, a study based on biobanks and databases from the IMPROVE study—a prominent European prospective cohort study comprising high-risk individuals who were initially free of clinically overt cardiovascular disease—concluded that IL-5 is not useful as a biomarker for CAD [36]. A recent study showed a decrease in serum concentrations of Th2 cytokines, such as IL-4, IL-13, and IL-5, in patients with CAD compared to healthy controls, with no significant differences between those with or without comorbid T2DM [37]. However, our study has found significantly elevated concentrations of all these cytokines in patients with CAD and comorbid T2DM. There are numerous reasons that could explain this discordance with the study by Madhumitha et al. [37]. For instance, the influence of other cardiovascular risk factors on the serum levels of these cytokines was not controlled for in their statistical analyses. Additionally, the diagnosis of CAD was based on the medical history of the participants and may not have been recent. Moreover, the mean age of the patients differed from our study (39 years for the CAD group and 61 years for the CAD and comorbid T2DM group). Finally, the determination of these cytokines was performed in blood rather than tears.

Recently, G-CSF levels in tears have been identified as a predictive factor for CAD in patients with suspected CAD [18]; however, once again, the number of studies exploring G-CSF as an inflammatory biomarker related to cardiometabolic diseases is limited. G-CSF plays a crucial role in the proliferation, differentiation, and maturation of neutrophils from peripheral blood progenitor cells, but T2DM impairs this mobilization. As a result, G-CSF has been used as a therapeutic intervention for treating diabetic foot ulcers and other related complications [38,39]. In the current investigation, the concentration of G-CSF was notably elevated in patients with coronary lesions and comorbid T2DM, which could be potentially associated with the restoration of immune cell mobilization. However, conversely, increased levels of G-CSF may also contribute to T2DM due to its insulin-desensitizing properties, as previously demonstrated in human muscle cells [40].

CCL11/eotaxin-1 is a CC chemokine that exhibits preferential activity on eosinophils but is secreted by a variety of cells, such as macrophages, lymphocytes, fibroblasts, smooth muscle endothelial cells, epithelial cells, and chondrocytes, among others. CCL11/eotaxin-1 is expressed at high levels at sites of vascular pathology and has been extensively studied in relation to cardiovascular events, including atherosclerosis and CAD. A first study showed elevated plasma concentrations of CCL11/eotaxin-1 in patients with CAD, and this chemokine was identified as an independent predictor of the angiographic extent of CAD [41]. On the other hand, the association between systemic levels of CCL11/eotaxin-1 and the risk of CAD was examined in the PRIME study—study with 10 years of follow-up of 9771 male participants—but it was not an independent predictor of coronary event [42]. In our previous study on tears, we found no significant differences in CCL11/eotaxin-1 concentrations between patients with CAD and those without [18]. To our knowledge, no studies have quantified this chemokine in tear samples from diabetic patients, although a systematic review and meta-analysis comparing CCL11/eotaxin-1 levels from various tissues and biological fluids in 621 patients with T2DM and 793 controls showed that diabetic patients had significantly higher concentrations of CCL11/eotaxin-1 than the controls [43]. Most of these studies used blood samples to determine CCL11/eotaxin-1, but none considered tear samples. There are a few studies that have measured CCL11/eotaxin-1 in eyes using vitreous fluid, but they focused on diabetic retinopathy and reported higher levels in patients with T2DM and retinopathy [44].

To the best of our knowledge, IL-5, G-CSF, and CCL11/eotaxin-1 concentrations emerge as inflammatory mediators associated with the coexistence of T2DM in patients with CAD for the first time. These discriminatory cytokines were obtained from tear samples using a rapid, safe, and non-invasive procedure, enabling us to establish distinct inflammatory profiles for patients with coronary lesions.

### Limitations and Future Perspectives

We acknowledge several limitations in our study, primarily related to its single-center design and sample size. Further research is needed to validate our findings in broader populations of patients with CAD, including larger sample sizes. This would enable the differentiation of CAD types (i.e., stable angina, STEMI, and NSTEMI) and allow for the assessment of age, sex, and all classical cardiovascular risk factors, in addition to T2DM, without statistical constraints. Although COPD and chronic kidney disease were not included in the multivariable analysis due to their low prevalence in our sample, their potential impact on ophthalmological parameters and/or tear inflammatory mediators cannot be ruled out. Additionally, future longitudinal studies are required to monitor changes in tear cytokine levels over time and to investigate their correlation with clinical characteristics during disease progression. The results of these future studies will be essential in establishing the role of these ophthalmological variables in the diagnosis and prognosis of patients with CAD, particularly in those with T2DM, from stages without symptoms of ocular complications.

On the other hand, in our study, not all of the evaluated inflammatory analytes could be quantified (i.e., IL-2, IL-10, IL-12p70, IL-15, and VEGF), which may be due to technical limitations or their absence in this fluid. Furthermore, it should be noted that the concentrations of prototypical pro-inflammatory cytokines, such as IL-1β and TNF-α, did not appear significantly affected by the presence of T2DM, in contrast to findings observed in other studies [45,46]. However, we must take into account that almost all of those studies are carried out on blood samples (i.e., serum and plasma) and with patients who may not have cardiovascular complications as they occur in our study. Therefore, it will be necessary to compare inflammatory alterations in both blood and tears in the same patients to determine any potential interactions between these two biological compartments—one systemic and the other more localized.

Finally, these assessments conducted on tear samples are not primarily intended to identify specific biomarkers for T2DM in the context of CAD, beyond a mere suggestion due to the limitations described. Instead, they unveil the presence of diverse inflammatory profiles in patients with CAD using non-invasive procedures incorporating ophthalmological variables. While the fundamental molecular and physiological mechanisms underlying these ophthalmologic alterations require further elucidation through subsequent studies, these findings are sufficiently relevant to warrant discussion within the context of cardiometabolic disorders.

## 5. Conclusions

This initial study demonstrates the usefulness of measuring specific ophthalmological variables, particularly cytokines from tear samples, in identifying a distinct inflammatory profile in patients with comorbid T2DM in the context of CAD. This differentiation, achieved through a non-invasive procedure, could serve as a valuable tool for evaluating, monitoring, and predicting coronary lesions, especially given the high prevalence of T2DM alongside other cardiovascular risk factors and the associated risk of bleeding complications.

Specifically, we observed significant decreases in ophthalmological parameters (i.e., MCV, MMT, RNFLT, and GCLT) and increases in tear concentrations of cytokines (i.e., IL-1ra, IL-4, IL-5, IL-6, IL-8, IL-9, IL-13, G-CSF, GM-CSF, IFN-γ, CCL3/MIP-1α, CCL4/MIP-1β, and CCL11/eotaxin-1) in patients with CAD and T2DM, with an inverse correlation among these variables.

Notably, among the ophthalmological variables, IL-5, G-CSF, and CCL11/eotaxin-1 concentrations were newly identified as tear inflammatory mediators predictive of the coexistence of T2DM after controlling for other prevalent cardiovascular risk factors (i.e., hypertension, hypercholesterolemia, obesity, smoking, sex, and age). These findings suggest that these tear cytokines could serve as a complementary tool for the stratification, diagnosis, and prognosis of patients with cardiometabolic conditions. However, their utility as biomarkers will need to be further investigated in future studies.

## Figures and Tables

**Table 1:** Baseline clinical characteristics of patients with CAD based on T2DM.

Variable			CAD		*p*-Value
		Total	Non-T2DM	T2DM	
Participants (n)		72	40	32	---
Sex, women [n (%)]		18 (25.0)	12 (30.0)	6 (18.8)	0.412 ^a^
Age (years) (mean ± SD)		60.7 ± 8.7	59.5 ± 7.9	62.4 ± 9.2	0.158 ^b^
Smoking habit [n (%)]		32 (44.4)	22 (55.0)	10 (31.3)	0.092 ^a^
Medical history and prior cardiovascular diseases [n (%)]	Hypercholesterolemia	25 (34.7)	10 (25.0)	15 (46.9)	0.081 ^a^
	Obesity	30 (41.7)	15 (37.5)	15 (46.9)	0.476 ^a^
	COPD	7 (9.7)	4 (10.0)	3 (9.4)	>0.999 ^a^
	Chronic kidney disease	5 (6.9)	2 (5.0)	3 (9.4)	0.650 ^a^
	High blood pressure	36 (50.0)	16 (40.0)	20 (62.5)	0.096 ^a^
	CAD	12 (16.7)	6 (15.0)	6 (18.8)	0.756 ^a^
	Peripheral artery disease	2 (2.8)	0 (0.0)	2 (6.3)	0.194 ^a^
	Stroke/TIA	3 (4.2)	1 (2.5)	2 (6.3)	0.582 ^a^
	Atrial fibrilation/arrhythmia	8 (11.1)	4 (10.0)	4 (12.5)	>0.999 ^a^
CAD type [n (%)]	Chronic CAD	24 (33.3)	13 (32.5)	11 (34.4)	0.983 ^a^
	STEMI	18 (25.0)	10 (25.0)	8 (25.0)	
	NSTEMI	30 (41.7)	17 (42.5)	13 (40.6)	
CAD severity [n (%)]	Non-significant lesions	18 (25.0)	11 (27.5)	7 (21.9)	0.267 ^a^
	Single-vessel disease	21 (29.2)	14 (35.0)	7 (21.9)	
	Multi-vessel disease	33 (45.8)	15 (37.5)	18 (56.3)	
Blood parameters at admission (mean ± SD)/[median (IQR)]	Albumin (g/dL)	3.3 ± 0.6	3.3 ± 0.8	3.2 ± 0.4	0.493 ^b^
	Creatinine (mg/dL)	0.98 (0.78–1.18)	1.01 (0.75–1.22)	0.96 (0.80–1.15)	0.891 ^c^
	Hemoglobin (g/dL)	13.9 ± 1.7	14.0 ± 2.1	13.6 ± 1.8	0.396 ^b^
	C-reactive protein (mg/L)	16.6 ± 8.5	14.7 ± 8.0	18.0 ± 9.3	0.110 ^b^
	eGFR (mL/min/1.73 m^2^)	78.25 (59.50–90.00)	79.00 (65.25–90.00)	77.50 (59.00–95.00)	0.724 ^c^
Treatment [n (%)]	Conservative	23 (31.9)	13 (32.5)	10 (31.3)	>0.999 ^a^
	PCI/CABG	49 (68.1)	27 (67.5)	22 (68.8)	

**^a^** *p*-values using the Fisher exact test. **^b^** *p*-values using the Student *t*-test. **^c^** *p*-values using the Mann–Whitney U test. Abbreviations: CABG = coronary artery bypass graft surgery; CAD = coronary artery disease; COPD = chronic obstructive pulmonary disease; eGFR = estimated glomerular filtration rate; IQR = interquartile range; NSTEMI = non-ST-elevation myocardial infarction; PCI = percutaneous coronary intervention; SD = standard deviation; STEMI = ST-elevation myocardial infarction; TIA = transient ischemic attack; T2DM = type 2 diabetes mellitus.

**Table 2:** Ophthalmological parameters in patients with CAD based on T2DM.

Ophthalmological Parameter	CAD		*p*-Value ^a,b^	Adjusted *p*-Value (*q*-Value) ^c^
	Non-T2DM	T2DM		
Number of eyes examined (n)	80	64	---	---
Intraocular pressure (mm Hg) (mean ± SD)	15.05 ± 2.95	14.08 ± 2.96	0.053 ^a^	ns
BCVA [median (IQR)]	1.00 (1.00–1.00)	1.00 (0.90–1.00)	0.029 ^b^	ns
Schirmer’s test (mm/5 min) [median (IQR)]	18.00 (12.00–28.75)	13.00 (9.00–26.00)	0.043 ^b^	ns
TCCT (µm) (mean ± SD)	550.55 ± 37.80	549.94 ± 38.36	0.925 ^a^	ns
PCCT (µm) (mean ± SD)	549.06 ± 42.21	554.02 ± 36.77	0.475 ^a^	ns
Axial length (mm) (mean ± SD)	23.66 ± 0.91	23.57 ± 1.57	0.722 ^a^	ns
CMT (µm) (mean ± SD)	269.08 ± 28.35	267.18 ± 28.56	0.696 ^a^	ns
MCV (mm^3^) (mean ± SD)	10.32 ± 0.51	10.02 ± 0.48	0.001 ^a^	0.008
MMT (µm) (mean ± SD)	286.51 ± 14.11	277.93 ± 13.42	>0.001 ^a^	0.004
RNFLT (µm) (mean ± SD)	92.65 ± 10.90	86.51 ± 12.21	0.002 ^a^	0.012
GCLT (µm) (mean ± SD)	81.59 ± 10.46	76.20 ± 11.74	0.005 ^a^	0.015
GCLMT (µm) (mean ± SD)	76.11 ± 17.94	69.62 ± 16.34	0.037 ^a^	ns
Choroidal thickness (µm) (mean ± SD)	306.95 ± 64.73	292.00 ± 82.28	0.247 ^a^	ns

**^a^** *p*-values using the Student *t*-test (with Welch’s correction when variances are unequal). **^b^** *p*-values using the Mann–Whitney U test. **^c^** Adjusted *p*-values (*q*-values) using the Benjamini-Hochberg procedure for controlling the FDR. Abbreviations: BCVA = best corrected visual acuity; CAD = coronary artery disease; CMT = central macular thickness; FDR = false discovery rate; GCLMT = ganglion cell layer minimum thickness; GCLT = ganglion cell layer thickness; IQR = interquartile range, MCV = macular cube volume; MMT = mean macular thickness; ns = non-significant; PCCT = pachymetry central corneal thickness; RNFLT = retinal nerve fiber layer thickness; SD = standard deviation; TCCT = topography central corneal thickness; T2DM = type 2 diabetes mellitus.

**Table 3:** Concentrations of inflammatory mediators in tears of patients with CAD based on T2DM.

Inflammatory Mediator	CAD		*p*-Value ^a^	Adjusted *p*-Value (*q*-Value) ^b^
	Non-T2DM	T2DM		
Number of eyes examined (n)	80	64	---	---
IL-1β (pg/mg) [median (IQR)]	0.305 (0.200–0.400)	0.269 (0.195–0.464)	0.842	ns
IL-1ra (ng/mg) [median (IQR)]	4.021 (3.102–6.076)	4.991 (4.268–6.565)	0.003	0.024
IL-4 (pg/mg) [median (IQR)]	0.273 (0.215–0.349)	0.364 (0.291–0.441)	<0.001	0.017
IL-5 (pg/mg) [median (IQR)]	0.900 (0.200–1.845)	3.508 (2.727–4.500)	<0.001	0.005
IL-6 (pg/mg) [median (IQR)]	0.818 (0.542–1.785)	1.614 (0.665–2.549)	0.005	0.026
IL-7 (pg/mg) [median (IQR)]	4.795 (3.359–5.893)	5.194 (3.456–6.888)	0.142	ns
IL-8 (pg/mg) [median (IQR)]	32.14 (22.60–62.33)	69.62 (41.41–120.17)	<0.001	0.012
IL-9 (pg/mg) [median (IQR)]	0.609 (0.020–4.208)	3.546 (0.510–6.397)	0.001	0.019
IL-13 (pg/mg) [median (IQR)]	0.292 (0.185–0.508)	0.404 (0.278–0.615)	0.008	0.029
IL-17A (pg/mg) [median (IQR)]	0.300 (0.300–0.600)	0.400 (0.300–0.600)	0.058	ns
TNF-α (pg/mg) [median (IQR)]	2.200 (1.000–7.380)	1.500 (1.000–2.500)	0.069	ns
G-CSF (pg/mg) [median (IQR)]	2.010 (0.073–9.893)	14.749 (7.165–22.429)	<0.001	0.007
GM-CSF (pg/mg) [median (IQR)]	0.459 (0.150–0.886)	0.567 (0.420–0.794)	0.011	0.031
IFN-γ (pg/mg) [median (IQR)]	52.33 (38.60–67.17)	62.93 (56.51–72.10)	0.001	0.021
PDGF-BB (pg/mg) [median (IQR)]	4.510 (1.510–15.150)	5.010 (1.635–7.510)	0.127	ns
CCL2/MCP-1 (pg/mg) [median (IQR)]	7.381 (3.188–19.015)	6.306 (2.979–21.156)	0.603	ns
CCL3/MIP-1α (pg/mg) [median (IQR)]	0.174 (0.128–0.338)	0.382 (0.212–0.490)	<0.001	0.014
CCL4/MIP-1β (pg/mg) [median (IQR)]	1.488 (0.731–2.489)	4.093 (2.404–5.660)	<0.001	0.010
CCL5/RANTES (pg/mg) [median (IQR)]	24.72 (11.12–46.38)	28.70 (20.25–49.21)	0.034	ns
CCL11/eotaxin-1 (pg/mg) [median (IQR)]	0.475 (0.354–0.629)	0.685 (0.635–0.861)	<0.001	0.002
CXCL10/IP-10 (ng/mg) [median (IQR)]	2.249 (1.445–3.100)	2.314 (1.771–3.501)	0.587	ns

**^a^** *p*-values using the Mann–Whitney U test. **^b^** Adjusted *p*-values (*q*-values) using the Benjamini-Hochberg procedure for controlling the FDR. Abbreviations: FDR = false discovery rate; G-CSF = granulocyte colony-stimulating factor; GM-CSF = granulocyte-macrophage colony-stimulating factor; IFN-γ = interferon gamma; IL = interleukin; IP-10 = interferon gamma-induced protein 10; IQR = interquartile range; MCP-1 = monocyte chemoattractant protein-1; MIP = macrophage inflammatory protein; ns = non-significant; PDGF-BB = platelet-derived growth factor-BB; RANTES = regulated on activation, normal T cell expressed and secreted; T2DM = type 2 diabetes mellitus.

**Table 4:** Logistic regression analysis using a backward method for distinguishing patients with CAD and comorbid T2DM from those patients without T2DM.

Explanatory Variable and Constant	B	Standard Error	Wald (dg)	*p*-Value	Exp^(B)^ (OR)	95% CI for OR	
						Lower	Upper
**Full Logistic Regression Model**							
MCV (mm^3^ × 100)	0.340	0.139	5.986 (1)	0.014	1.405	1.070	1.846
MMT (µm)	−1.255	0.506	6.158 (1)	0.013	0.285	0.106	0.768
IL-4 (pg/mL × 10)	−0.856	0.382	5.026 (1)	0.025	0.425	0.201	0.898
IL-5 (pg/mL)	0.737	0.193	14.518 (1)	<0.001	2.090	1.430	3.053
IL-6 (pg/mL)	−0.148	0.143	1.064 (1)	0.302	0.863	0.652	1.142
IL-8 (pg/mL)	0.008	0.007	1.357 (1)	0.244	1.008	0.995	1.021
IL-9 (pg/mL)	−0.304	0.200	2.312 (1)	0.128	0.738	0.499	1.092
IL-13 (pg/mL × 10)	0.386	0.151	6.536 (1)	0.011	1.471	1.094	1.977
G-CSF (pg/mL)	0.362	0.102	12.636 (1)	<0.001	1.436	1.176	1.753
GM-CSF (pg/mL)	−0.011	0.194	0.003 (1)	0.955	0.989	0.676	1.447
CCL3/MIP-1α (pg/mL × 10)	−0.887	0.370	5.736 (1)	0.017	0.412	0.199	0.851
CCL4/MIP-1β (pg/mL)	0.073	0.228	0.101 (1)	0.751	1.075	0.687	1.683
CCL11/eotaxin-1 (pg/mL × 10)	0.986	0.260	14.335 (1)	<0.001	2.680	1.609	4.465
Constant	0.232	6.820	0.001 (1)	0.973	1.261	---	---
**Reduced logistic regression model (Backward method: Wald)**							
IL-5 (pg/mL)	0.519	0.146	12.617 (1)	<0.001	1.680	1.262	2.237
G-CSF (pg/mL)	0.062	0.022	8.164 (1)	0.004	1.064	1.020	1.109
CCL11/eotaxin-1 (pg/mL × 10)	0.444	0.139	10.279 (1)	0.001	1.560	1.189	2.046
Constant	−4.934	0.939	27.610 (1)	<0.001	0.007	---	---

Abbreviations: B = coefficient for the constant (intercept); CI = confidence interval; dg = degrees of freedom for the Wald chi-square test; G-CSF = granulocyte colony-stimulating factor; GM-CSF = granulocyte-macrophage colony-stimulating factor; IL = interleukin; OR = odds ratio; MCV = macular cube volume; MIP = macrophage inflammatory protein; MMT = mean macular thickness.

## Data Availability

The datasets used and/or analyzed during the current study are available from the corresponding author upon reasonable request.

## Supplementary Materials

The following supporting information can be downloaded at: https://www.mdpi.com/article/10.3390/jcm13164906/s1. Table S1: Cardiovascular risk factors of patients with CAD suspicion based on the confirmation of CAD; Table S2: Logistic regression and ROC analyses for distinguishing patients with CAD and comorbid T2DM from those patients without T2DM using IL-5, G-CSG, or CCL11/eotaxin-1 concentrations as explanatory variables.
