# Supplementary material for: Differential Ophthalmological Profile in Patients with Coronary Artery Disease Coexisting with Type 2 Diabetes Mellitus: Elevated Tear Cytokine Concentrations
Source: J Clin Med. 2024 Aug 20;13(16):4906. doi: 10.3390/jcm13164906 (PMC11355890; doi:10.3390/jcm13164906)
Supplement: Supplementary file 1 [file jcm-13-04906-s001.zip › New Table S1.pdf]

Table S1. Cardiovascular risk factors of patients with CAD suspicion based on the confirmation of CAD.

Variable	CAD suspicion			P-value
	Total	Non-CAD	CAD	
Participants (n)	100	28	72	---
Sex, women [n (%)]	27 (27.0)	9 (32.1)	18 (25.0)	0.412 [a]
Age (years) (mean ± SD)	60.3 ± 9.0	59.3 ± 9.7	60.7 ± 8.7	0.467 [b]
Smoking habit [n (%)]	40 (40.0)	8 (28.6)	32 (44.4)	0.176 [a]
T2DM [n (%)]	43 (43.0)	11 (39.3)	32 (44.4)	0.661 [a]
Hypercholesterolemia [n (%)]	36 (36.0)	11 (39.3)	25 (34.7)	0.817 [a]
Obesity [n (%)]	42 (42.0)	12 (42.9)	30 (41.7)	>0.999 [a]
COPD [n (%)]	9 (9.0)	2 (7.1)	7 (9.7)	>0.999 [a]
Chronic kidney disease [n (%)]	5 (6.9)	2 (7.1)	5 (6.9)	>0.999 [a]
High blood pressure [n (%)]	39 (39.00)	13 (46.43)	36 (50.00)	0.825 [a]

([a]) P-values using the Fisher exact test. ([b]) P-values using the Student t test.

Abbreviations: CAD = coronary artery disease; COPD = chronic obstructive pulmonary disease; SD = standard deviation; T2DM = type 2 diabetes mellitus.
