# Supplementary material for: Differential Ophthalmological Profile in Patients with Coronary Artery Disease Coexisting with Type 2 Diabetes Mellitus: Elevated Tear Cytokine Concentrations
Source: J Clin Med. 2024 Aug 20;13(16):4906. doi: 10.3390/jcm13164906 (PMC11355890; doi:10.3390/jcm13164906)
Supplement: Supplementary file 1 [file jcm-13-04906-s001.zip › New Table S2.pdf]

Table S2. Logistic regression and ROC analyses for distinguishing patients with CAD and comorbid T2DM from those patients without T2DM using IL-5, G-CSG, or CCL11/eotaxin-1 concentrations as explanatory variables.

	Logistic regression analysis							ROC analysis				
**Explanatory variable and constant**	**B**	**Standard error**	**Wald (dg)**	**P-value**	**Exp(B) (OR)**	**95%CI for OR**		**AUC**	**Standard error**	**P-value**	**95%CI for AUC**	
						**Lower**	**Upper**				**Lower**	**Upper**
IL-5 (pg/mL)	0.755	0.128	34.745 (1)	<0.001	2.127	1.655	2.754	0.823	0.037	<0.001	0.750	0.897
Constant	-2.057	0.371	30.784 (1)	<0.001	0.128	---	---					
G-CSF (pg/mL)	0.086	0.020	18.822 (1)	<0.001	1.089	1.048	1.132	0.809	0.037	<0.001	0.736	0.881
Constant	-1.224	0.273	20.089 (1)	<0.001	0.294	---	---					
CCL11/eotaxin-1 (pg/mL×10)	0.654	0.124	28.013 (1)	<0.001	1.923	1.510	2.451	0.817	0.035	<0.001	0.748	0.885
Constant	-4.241	0.785	29.190 (1)	<0.001	0.014	---	---					

Abbreviations: AUC = area under the curve; B = coefficient for the constant (intercept); CI = confidence interval; dg = degrees of freedom for the Wald chi-square test; G-CSF = granulocyte colony-stimulating factor; IL = interleukin; OR = odds ratio; ROC = receiver operating characteristic.
